# Prognosis conversations in advanced liver disease: A qualitative interview study with health professionals and patients

**DOI:** 10.1371/journal.pone.0263874

**Published:** 2022-02-18

**Authors:** Jennifer Arney, Caroline Gray, Jack A. Clark, Donna Smith, Annie Swank, Daniel D. Matlock, Jennifer Melcher, Fasiha Kanwal, Aanand D. Naik

**Affiliations:** 1 VA Health Services Research & Development Center for Innovations in Quality, Effectiveness, and Safety, Michael E. DeBakey VA Medical Center, Houston, Texas, United States of America; 2 Department of Sociology, University of Houston-Clear Lake, Houston, Texas, United States of America; 3 Center for Innovation to Implementation, VA Palo Alto Health Care System, Palo Alto, California, United States of America; 4 Department of Health Policy and Management, Boston University School of Public Health, Boston, Massachusetts, United States of America; 5 Department of Medicine, Section of Health Services Research, Baylor College of Medicine, Houston, Texas, United States of America; 6 Division of Geriatric Medicine, Department of Medicine, University of Colorado School of Medicine, Aurora, Colorado, Colorado; 7 Department of Medicine, Section of Gastroenterology and Hepatology, Baylor College of Medicine, Houston, Texas, United States of America; 8 Department of Medicine, Section of Geriatrics and Palliative Medicine, Baylor College of Medicine, Houston, Texas, United States of America; 9 Department of Management, Policy, and Community Health, School of Public Health, University of Texas Health Science Center, Houston, Texas, Colorado; University of Technology Sydney, AUSTRALIA

## Abstract

Advanced Liver Disease (AdvLD) is common, morbid, and associated with high likelihood of death. Patients may not fully understand their prognosis and are often unprepared for the course of illness. Little is known about how and when to deliver prognosis-related information to patients with AdvLD, who should participate, and what should be discussed. We conducted in-depth interviews with a multi-profession sample of Hepatology clinicians and patients with AdvLD. Participants were drawn from three geographically diverse facilities (New England, Texas, California). We used inductive and deductive qualitative data analysis approaches to identify themes related to AdvLD prognosis discussions. Thematic analysis focused on content, timing, and participants’ roles in prognosis discussions. In total, 31 patients with AdvLD and 26 multi-profession clinicians completed interviews. Most participants provided a broad conceptualization of prognosis beyond predictions of survival, including expectations about illness course, ways to manage or avoid complications and a need to address patients’ emotions. Patients favored initiating discussions early in the AdvLD course and welcomed a multi-profession approach to conducting discussions. Clinicians favored a larger role for specialty physicians. All participants recognized that AdvLD prognosis discussions occur infrequently and favored a structured, standardized approach to broadly discussing prognosis. Patients with AdvLD and their clinicians favored a multifaceted approach to prognosis conversations including discussions of life expectancy, predictions about likely course of liver disease, and expected changes in function and capabilities over time. Structured and early prognosis discussions should be part of routine AdvLD care.

## Introduction

Advanced liver disease (AdvLD), defined as cirrhosis of any cause with one or more liver-related complications, is a highly morbid condition characterized by frequent complications and limited survival [1, 2]. The median survival for AdvLD is about 60% at one-year and 50% at two-years (85% Childs-Pugh A to 35% Childs-Pugh C) [2]. Patients with AdvLD face multiple hospitalizations, declining functional status, and worsening symptoms that culminate in significant burden of illness over the last months of life [3]. As with other serious illnesses, optimal care for patients with AdvLD involves close attention to patients’ values, emotions, and concerns, and shared decisions about self-management and treatment preferences [1, 4].

Prior studies describe the course of AdvLD as fraught by pervasive, enduring, and universally shared uncertainty resulting in multidimensional distress. While conversations between patients and their clinicians about the course and prognosis of illness may mitigate this distress, they often come late in the course of cirrhosis care [5, 6]. Prior studies demonstrate that primary care clinicians and patients are often reluctant to discuss prognosis and defer these conversations to liver specialists [7, 8]. However, Low et al. [5] describe how liver specialists have difficulties initiating discussions regarding prognosis, planning for potential deterioration, often have limited knowledge of palliative care and advanced care planning.

Efforts to improve prognosis conversations exist for cancer, end-stage renal disease, and other serious illnesses [9–11]. Interventions to enhance such conversations can result in decreased anxiety and distress, reduce current treatment burden, and add fewer unwanted or low-value interventions [12, 13]. A central element to all such conversations is the discussion of prognosis, most often framed as time-based predictions and informed by clinical algorithms that estimate the probability of mortality in the near term. Child-Turcotte-Pugh and Model for End-Stage Liver Disease (MELD) scores serve this purpose in the context of AdvLD. Effective prognosis discussions should also include functional (i.e., outcomes that matter to patients) prognosis, acknowledge uncertainty of estimates, and present treatment options aligned with patient values, goals, and preferences [11]. Validated tools such as the Supportive and Palliative Care Indicator Tool (SPICT) [14] and the Bristol Prognostic Screening Tool (BPST) [15] can also help guide clinicians to identify patients with unmet specialist palliative care needs who would benefit from early referral for prognosis discussions due to potential deterioration [16].

Little is known about the nature and specific barriers to discussions of prognosis within this broader framework for patients with AdvLD [6]. To address this gap, we conducted a qualitative study with in-depth interviews of patients with AdvLD and a multi-profession sample of clinicians with expertise in liver disease care, drawn from three geographically diverse Veterans Affairs (VA) medical centers. The aims of the study were to describe what participants believe a prognosis discussion should entail, when during the course of illness the discussions should occur, who should participate and lead the conversations, and how information is delivered.

## Materials and methods

### Study design and setting

We conducted one-on-one, in-depth qualitative interviews with 26 clinicians in hepatology specialty care and 31 patients with advanced liver disease, sampled from VA healthcare systems in Southeast Texas, Northern California, and New England. All 3 sites include liver tumor board. Only one site (Southeast Texas) offers transplant services. Two sites (Northern California and New England) refer to nearby VA regional transplant centers that provide transportation and lodging for the Veteran and 1 caregiver.

Clinicians were referred by local chiefs of hepatology and gastroenterology from current clinic staffing structures. Twenty-six out 33 referred clinicians responded and completed interviews. No payment was provided.

Patients were recruited from a central location via opt-out mailings. We used a population screening approach of eligibility (inclusion and exclusion) criteria from a computer-generated randomized list programmatically identified in the EMR for each VA site. Patient inclusion criteria were age 18 and over with ICD-9/10 codes for cirrhosis, its complications (ascites, varices with or without bleeding, spontaneous bacterial peritonitis) or filled prescriptions for medications used to treat these complications (spironolactone, rifaximin, lactulose) [17–19]. Exclusion criteria were recent chart notes indicating overt hepatic encephalopathy, those who were currently hospitalized, or in skilled nursing or hospice care. Out of 100 invitation letters mailed, 31 patients enrolled and completed interviews. Letters invited patients to participate in a one-time, one-on-one telephone interview to discuss experiences living with advanced liver disease and care they’ve received. Each patient participant received a $40 payment. Recruitment was stopped at the point of thematic saturation, defined as the point when two independent coders agreed that no new thematic concepts emerged from subsequent interviews [20]. Interviews with clinicians took place from October 2018 through August 2019 and patient interviews from January 2019 to November 2019. Interviews with both clinicians and patients averaged 30 minutes to an hour, with patient interviews tending to last longer. All interviews were conducted via telephone. With participants’ permission, interviews were audio recorded and transcribed.

This study was approved by the Institutional Review Board (IRB) for Baylor College of Medicine and Affiliated Hospitals/Houston VA (H-33191), IRB for Stanford University/VA Palo Alto Health Care system (eProtocol #42849), and the VA Connecticut Healthcare System, Human Research Protections Program (MIRB# 02183). Oral consent was obtained for all participants. Written consent was not obtained because the act of obtaining written consent would create paper documentation that would pose a greater risk of breach of privacy. We obtained verbal informed consent twice–prior to recording and then after recording started for documentation.

### Data collection

Clinicians and patients were asked about their experiences with and perceptions of AdvLD care, specifically communication strategies and discussions of prognosis and disease trajectory. Interview guides were based on the integrated model of advanced liver disease, which specifies the need to develop more informed techniques for eliciting patients’ beliefs, goals, and preferences, as well as processes for identifying clinicians’ clinical preferences and their awareness of patients’ needs [1]. Toward this end, our interview guides were designed to elicit how patients and clinicians understand advanced liver disease, and their preferences regarding communication about disease severity, prognosis and trajectory. These goals were communicated to participants at the beginning of the interviews. Following interviews, participants were not contacted again.

Interview guides were revised throughout the interviewing process to reflect emergent findings and clarify developing areas of interest (S1 Appendix). Content in earlier interviews informed areas of inquiry and probing in subsequent interviews. All interviews were included in the analyses. Two medical sociologists (JA and CG) trained in qualitative methods conducted all interviews. Both interviewers are Caucasian females in their early 40’s with Ph.D.’s in Sociology. Both have extensive experience conducting qualitative interviews with clinicians and patients, analyzing qualitative data, and presenting qualitative findings. During data collection and data analysis, the qualitative interviewers met weekly with the entire multidisciplinary research team to discuss progress. Meetings included discussion of interview field notes, interview themes and early analysis, as well as the interviewers’ own experiences, beliefs, and potential biases. This reflexivity allowed the researchers to minimize effects of biases and enhance the credibility and rigor of the findings [21]. While one researcher (JA) could be considered a complete outsider to advanced liver disease, having neither experienced the disease or been a caregiver for a patient with the disease, one researcher (CG) had a family member affected by advanced liver disease, giving her more of an insider’s position. Since both, insiders and outsiders, bring strengths and weaknesses to studying a topic [21], we maintain that this unique combination of an insider and an outsider benefitted our research and facilitated deeply reflexive conversations as the study unfolded [22].

### Data analysis

Analysis of clinician and patient interviews occurred at separate time points and both involved inductive and deductive approaches to identify themes related to AdvLD prognosis discussions. After receiving several transcripts from each group of interview participants–clinicians and patients—several members of the analytic team reviewed them in their entirety, created memos, and using thematic analysis, created a preliminary codebook. Codes were anchored in interview guide questions as well as emergent findings [23]. Codebooks were piloted and revised with additional codes added as necessary. Two team members independently coded all transcripts (JA and CG), and a third coder performed secondary coding to ensure accuracy and credibility of code assignments (AS) [22]. All coding was performed using Atlas.ti (version 8.2). After initial coding, all codes were collapsed into broader categories, which team members then summarized, highlighting themes that spanned across interviews within each interview sample [24]. Lastly, the team identified parallels across interviews with clinicians and patients, comparing and contrasting clinician and patient perceptions around communication between patients and clinicians, descriptions of prognosis discussions or lack thereof, and patient and clinician perceptions of needs as liver disease progresses. After coding was completed, the full study team participated in the integration of codes into themes describing the content, process, and participants of prognosis discussions for AdvLD. Findings pertaining to other themes related to the integrated model of advanced liver disease will be reported in additional manuscripts.

## Results

We conducted semi-structured interviews with 26 clinicians and 31 veterans with advanced liver disease (see Table 1). The sample of clinicians included 20 women and 6 men. Multiple professions were represented in our sample, including Gastroenterology, Hepatology, and Transplant Physicians (34.6%), Gastroenterology Physician Assistants (7.7%), Gastroenterology/Hepatology nurses and nurse practitioners (27%), social workers and psychologists (11.5%), palliative care providers (15.4%) and pharmacists (3.8%). Patients were all men and represented a diversity of racial and ethnic backgrounds. Patients ranged in age from 54 to 87 years, with a mean of 67 years. Most patients (74.1%) had high school or some college education. Half of our respondents (51.6%) were married. Roughly half (45.2%) reported 4–6 comorbidities [25]. Most patients were classified as Child-Pugh Class A (80.6%).

**Table 1:** Demographic characteristics of the study population.

Patients (n = 31)	Value	
**Age in years**, mean (range)	67 (54–87)	
**Race and Ethnicity, number (percentage)**		
African American	7 (22.6)	
White, non-Hispanic	17 (54.8)	
Hispanic	3 (9.6)	
Other	2 (6.5)	
No Response	2 (6.5)	
**Education Completed, number (percentage)**		
Less than High School Grad	2 (6.5)	
High School graduate or some college	23 (74.1)	
College graduate	4 (12.9)	
No Response	2 (6.5)	
**Annual Income, number (percentage)**		
<$20,000	10 (32.3)	
$20,000 –$50,000	14 (45.1)	
>$50,000	2 (6.5)	
Don’t Know or Refused	5 (16.1)	
**Marital Status, number (percentage)**		
Married	16 (51.6)	
Widowed, Separated or Divorced	9 (29)	
Never married	4 (12.9)	
No Response	2 (6.5)	
**Comorbidities per Patient, number (percentage)**		
2–3	4 (12.9)	
4–6	14 (45.2)	
7–9	8 (25.8)	
10–11	3 (9.6)	
No Response	2 (6.5)	
**Comorbid Conditions** * **, number (percentage)**		
High blood pressure	22 (71)	
Arthritis or any kind of rheumatism	20 (64.5)	
Chronic neck, back or spine troubles	18 (58)	
Depression and/or Anxiety	24 (77.4)	
Cancer	13 (41.9)	
Diabetes	12 (38.7)	
Heart disease	9 (29)	
Kidney, Stomach and/or Bladder trouble	26 (83.9)	
Migraines	7 (22.6)	
Other mental health issues	7 (22.6)	
Anemia	5 (16.1)	
Asthma, Bronchitis or Lung disease	9 (29)	
Stroke	3 (9.6)	
Repeated seizures	1 (3)	
No Response	2 (6.5)	
**Child-Pugh Severity Level**, number (percentage)		
A	25 (80.6)	
B or C	6 (19.4)	
**Healthcare Providers (n = 26)**	Value	
**Clinical Role,** number (percentage)		
Gastroenterology, Hepatology, or Transplant Physicians	9 (34.6)	
Gastroenterology Physician Assistants	2 (7.7)	
Gastroenterology/Hepatology nurses and nurse practitioners	7 (27)	
Social Workers & Psychologists	3 (11.5)	
Palliative Care Providers	4 (15.4)	
Pharmacists	1 (3.8)	
**Gender,** number (percentage)		
Women	20 (77)	
Men	6 (23)	

*Reflects the number of patients who self-reported having that condition.

Analysis revealed three themes pertaining to prognosis conversations: (1) components of prognosis discussions, (2) timing of discussions, and (3) roles and responsibilities. Each theme and related subthemes are as follows.

### (1) Components of prognosis discussions

#### Content of discussions

Clinicians and patients held differing beliefs about what prognosis discussions should entail. For some clinicians, the term prognosis denoted references to mortality and estimates of survival time. For others, prognosis discussions encompassed substantially more. For example, discussions might begin with an explanation of the patient’s diagnosis and the severity of their illness, along with key clinical terms such as “compensated” and “decompensated” illness to describe the likely trajectory of AdvLD. Prognosis discussions could include what patients might expect in the future, along with the ways to manage the disease, potential causes of their disease, and the importance of avoiding risk factors.

*“Usually the first impression is try to let them understand the potential cause or causes of their liver disease. Once they understand it, I let them know cirrhosis is a serious but manageable condition. It’s serious in the case that it can progress, that by removing the cause of their liver disease most of the damage to the liver can get better, and if it’s too far gone that we cannot reverse the scar tissue, we can also decrease the risk of complications. So once a patient understands the severity of their liver disease, the potential complications, and ways to manage it and prevent further decompensation, I think I’m done with my job.” (Clinician #25, Physician Assistant, Female, 7 years experience in liver disease care)*

While a few patients were satisfied with the information they’ve received about their liver disease, many patients expressed preference for additional information. In particular, patients desired information beyond expected survival time, including potential complications and how the disease could affect their quality of life in the future. For instance, patients wondered how disease progression might affect their mobility or require major dietary changes.

*“It would be nice to know that, okay I’m having problems getting out of bed in the morning, and that’s only going to get worse…So my appetite is going to go down…or I’ll only be able to take liquids, as opposed to solids…If they have some information that…[will tell me] what’s going to happen towards the end. You’re going to not be able to keep food down, you’re not going to be able to drive. . . .” (Patient #1, 82-year-old White Male)*

Another patient similarly expressed a desire for more information about the nature and progression of liver disease:

*“The more I think about it*, *the more uncertain I am about what to expect*. *They can say maybe something will evolve in the next six months to a year*. *They can give me a timeline on when things will start to affect my day-to-day activity… Am I at a certain stage*?…*And I don’t have any symptoms now…Does that mean I’m okay*? *And is there going to be a point where they will evolve*? *Can you give me some indication*? *Does it move fast*? *Does it move slow*? *Does it vary with an individual*? *Is there something I can do in the way of diet or anything I can do to help keep it from being more active*? *And there are some things that they may know that I haven’t been told yet*, *like eat more salads*, *more fruits or something…So*, *yeah*, *like I say*, *I’m sort of in the dark*.” *(Patient #2*, *76-year-old White Male)*

As the quotes suggest, patients indicated a desire for information that could help them prepare for the future and participate in self-care.

Patients may be shocked to learn details of their liver disease and its future course. Their reactions may also shift as they live with the disease. For example, one patient indicated that though it was initially difficult to learn about his liver disease, he was able to move on:

*“I found it was initially very devastating, but I dealt with it for a few days, and you put things in their place because you have to in order to move on.” (Patient #11, 61-year-old White Male)*

Initially, patients may feel anxiety from talking about prognosis; however, according to patients, withholding prognosis information did not allay patients’ anxiety in the long term. Discussing prognosis may mitigate anxiety by reducing uncertainty, as the following quote from a patient demonstrates:

*“I don’t know how bad off I am right now, if it’s like 10 percent of my liver is affected or if it’s 80 percent of my liver that’s affected. I don’t know exactly where I am and that’s the part that worries me, because I don’t know if they don’t want to tell me because it’s so bad, or they don’t want to tell me because they’re not that worried about it.” (Patient #25, 71-year-old White Male)*

Another patient similarly indicated that knowing about his prognosis might decrease the anxiety caused from “fear of the unknown.” Ultimately, patients suggested that prognosis information may empower them to more effectively participate in self-care and to plan for their future.

“*Knowledge is power…The more that I know the better off I feel I am*.” *(Patient #16*, *54-year-old White Male)*.

#### Using clinical tools to present prognosis

Prognosis discussions contain a mixture of numeric and descriptive components, as clinicians use numbers that must be explained and rendered personally relevant to patients. Discussions may begin with a formal risk score like MELD, along with survival ranges and population averages as a starting point. While some clinicians do not revisit risk scores at every encounter, others indicated that their patients want to monitor and discuss changes in their risk scores at every encounter.

*“[Patients are] actually down to earth [and] really want to know numbers … because you have the MELD score and all these other scores we [are] able to tell them numbers… Most of my patients already know their MELD they follow it and they want to know.” (Clinician #7, Transplant Hepatologist, Female)*

When presenting prognostic information, some clinicians do point out the limitations of risk scores.,

*“I think one way to kind of make things a little smoother is to preface that I’m not psychic, I am not God and this is an estimation. But based on the data I’m looking at, based on my experience with other patients with this disease, this is what I foresee.” (Clinician #24, APRN, Palliative Care, Female, 3 years experience in liver disease care)*

This sets the stage for a personalized discussion about an individual patient’s health status and recommendations for self-care.

Some patients confirmed that they find MELD scores useful. Liver transplant candidates in particular are keenly aware of their MELD score; they monitor changes in the score and even anticipate how it will change in the coming months as they await a transplant.

*“That’s one of the things I have to ask, because nobody ever tells me what my MELD score is, unless I ask… It helps me gauge where I’m at.” (Patient #22, 61-year-old White Male)*

Other patients did not understand or track risk scores and survival statistics presented by their clinicians.

*“I don’t know the numbers ma’am. They give me a chart, but that thing, I look at it, you know, and I don’t understand it that much. So I don’t worry about it.” (Patient #27, 64-year-old African American Male)*

The variability in patients’ information preferences and needs illustrates the importance of providing information in various formats and checking understanding.

In addition to discussing formal risk scores, clinicians recommended providing patients with written materials to take home. Some clinicians expressed the desire for additional media to support prognosis conversations, including PowerPoints, models depicting liver disease, and even educational videos that can be shared in the clinic.

Patients indicated they find written materials useful and they voiced a need for more personalized information to help them understand and manage their disease and prepare for the future. One patient described his desire for written information that is more personalized:

*“They got like a hundred pamphlets of that stuff already printed out, stapled up, and they just hand it to everybody. And I’m sure that’s all the basics, but I mean really, I’d rather have something I can go home, that I’m reading kind of about me and what my condition is and any way that I can improve on what I’m doing to try to get me a little bit better.” (Patient #4, 63-year-old White Male)*

#### Communication approaches

Clinicians described techniques they use to facilitate more comfortable and less anxiety-provoking prognosis discussions. For instance, clinicians might share with patients the need to discuss “difficult news” rather than “bad news.” Within the conversation, they might avoid trigger words like “end-stage liver disease” and instead refer to this stage as “cirrhosis with complications.” Some clinicians avoid the terms “hospice” and “palliative care” when suggesting supportive care to patients with liver disease.

Some clinicians aim to facilitate flexible discussions with frequent check-ins to make sure they are meeting patients’ needs. This patient-centered approach involves setting goals, clarifying preferences, and aligning care plans to achieve patients’ priorities, and ultimately aiming to empower patients. One clinician described a patient-led approach:

*“Well, first I ask them if they want to know. And if they want to know, how much do they want to know. Do they want to know in broad strokes? Do they want to know specific details? Again, empowering them.” (Clinician #23, Liver Transplant Social Work Coordinator, Female, 5 years experience in liver disease care)*

Another clinician suggested a survey to elicit patient’s information needs prior to the medical encounter. Others described a flexible approach to presenting information, with a focus on taking cues from the patients on when to delve deeper.

*“Sometimes you can start the conversation and it needs to go a very different way in order to meet that patient’s needs at the time. And then discuss it at a follow-up visit or maybe even on a phone call.” (Clinician #13, APRN Liver Clinic, Female, 20 years experience in liver care)*

Patients’ communication preferences pertained more to content, rather than style. However, some patients did indicate a preference for honest and straightforward communication from their providers. Specifically, patients indicated that they don’t want clinicians to “candy coat” or “sugar coat” what might be considered difficult news.

*“The only thing I don’t want them to do is candy coat it… I mean, I’m a big boy…Physically and mentally I’m a big boy.” (Patient #16, 55-year-old White Male)*

### (2) Timing of prognosis discussions

#### Early prognosis discussions

Some clinicians noted that prognosis should be discussed upon initial diagnosis, even when information “sounds shocking,” as one hepatologist put it. Prognosis discussions with compensated patients often highlight potential complications and the unpredictable nature of liver disease.

*“I try to impress upon them that while everything is good now … it has the potential to get worse. …. And even though things are good now, we want to keep them that way and we want to keep it where they don’t have to come to the emergency room or be admitted to the hospital.” (Clinician #24, Gastroenterology Physician, Female, 3 years experience in liver care)*

Though preferring early prognosis discussion, some clinicians avoided these on the first visit. Particularly with compensated patients, these clinicians noted that they prefer to build a therapeutic relationship with patients prior to introducing prognosis information.

Patients often expressed a desire for early and information-rich prognosis conversations. One patient acknowledged feeling “in the dark” and expressed dissatisfaction that his hepatologist did not discuss prognosis prior to referring him to oncology. At that point, he had many questions he felt should have been addressed when he began care in hepatology.

#### Delayed prognosis discussions

Some clinicians indicated that they rarely discussed prognosis or altogether avoid the topic with compensated patients whose liver disease is stable. Clinicians may not discuss prognosis with compensated patients if the diagnosis has no bearing on immediate treatment decisions and if they think that such patients are likely to die from something else. Clinicians also prefer to delay prognosis conversations in order to maintain patients’ hope and avoid a depressed mood, particularly with patients who are early in the disease course. Clinicians tended to filter information and use language instrumentally in effort to keep patients hopeful and engaged in care.

*“I want them to have hope, and I feel like having those conversations could affect that.” (Clinician #21, Gastroenterology Physician, Female, 19 years experience in liver care)*

Similarly, one clinician explained why she dampens her language during prognosis discussions:

*“I think my main fear is that I will send someone into a worse depression… [and] trigger some sort of suicidal ideation, hopelessness…. that’s probably my main concern when I have these discussions with them, which is probably why I’m always dampening it.” (Clinician #3, Nurse Practitioner, Female, 5 years experience in liver care)*

Clinicians viewed decompensation as a turning-point in the course of patients’ liver disease. There was general agreement among clinicians that decompensation is a time at which discussion of prognosis should always take place. Other clinicians who favored earlier prognosis discussions tended to follow-up on those previous conversations and revisit prognosis estimates after patients developed decompensation. Whereas prognosis discussions with compensated patients might be broad in nature, conversations with decompensated patients and their caregivers seem to focus on mortality and survival data, particularly for very advanced patients. For example, one clinician described a scenario where survival estimates become immediately necessary:

*“If I’m seeing somebody for instance in the intensive care unit and families don’t always ask [about prognosis]. Like if their loved one is unconscious or whatever the case may be. But…if they are in their final hours or days we do tell [family members], because we want to make sure they have whoever they want to have here present when their loved one dies.” (Clinician #24, APRN Palliative Care, Female, 3 years experience in liver care)*

While many patients indicated a preference for early prognosis conversations, some expressed no such preference and simply defer to their clinicians to provide information when it is necessary.

*“I like to cross my bridges when I come to them…Maybe have [prognosis information] as a handout or a packet of some kind, that at some point in the patient’s disease progression, you deem it appropriate to dispense the information.” (Patient #11, 61-year-old White Male)*

### (3) Roles and responsibilities in prognosis conversations

Interviews revealed a lack of role clarity around initiating and carrying out prognosis conversations. However, there was consensus among liver clinic clinicians, clinical pharmacists, clinical psychologists, transplant team members (social work coordinator, liver transplant coordinator, liver transplant case manager), and palliative care providers that the patient’s Hepatologist or Gastroenterologist should play a prominent, if not lead, role and that they have a responsibility to introduce prognosis information. Other clinicians expressed willingness to follow up on prognosis discussions if patients have questions or if they need psychosocial and emotional support.,

*“I’m not the one to initiate that discussion. The medical providers would probably do that. But it does come up at times…I can’t make a judgement at that so I always…encourage them to ask the medical providers or talk about like, have you asked what your prognosis is? What would that mean for you if you didn’t have that much time left?” (Clinician #4, Clinical Psychologist, Female)*

Other team members described team processes to ensure that patients have support available after a prognosis discussion, including making available palliative care social workers.

Non-physician clinicians may defer prognosis conversations to attending physicians, liver fellows, or APRNs. If the patient is a candidate for transplant, clinicians sometimes defer the conversation to the transplant team. Finally, some clinicians believe the responsibility for prognosis conversations falls on the palliative care providers. However, palliative care providers noted that they assume patients have already discussed prognosis before coming to palliative care. This lack of role clarity may lead to delays in prognosis conversations.

*“Sometimes I’ve seen notes, patient with cirrhosis, decompensated cirrhosis. It’s not even mentioned in the progress note, which means it’s not even addressed.” (Clinician #10, Physician Assistant, Female, 7 years experience in liver disease care)*

Clinicians believed Hepatologists have longer relationships with patients, greater rapport, and are better situated to engage patients in such conversations. Hepatologists generally recognized that discussing survival estimates and disease progression is their responsibility. Some Hepatologists acknowledged discomfort discussing risk of death and desired additional training to do it better. Due to this discomfort, some admitted that they do not regularly discuss prognosis with their patients.

*“I think, don’t talk about [dying from complications] because I’m trying to get them to liver transplantation.” (Clinician #21, Gastroenterology Physician, Female, 19 years experience in liver care)*

According to patients, Hepatologists do not necessarily need to deliver prognosis information. Patients are open to discussing prognosis with another clinician on their care team who is available to take time to give them accurate information and answer their questions.

*”A nurse, yeah. Just somebody that could sit down and spend some time with you…it’s a little bit more comforting, you know.” (Patient #25, 71-year-old White Male)*

## Discussion

Clinicians with expertise in liver disease care and patients with AdvLD described the multifaceted nature of prognosis discussions for AdvLD. Participants conveyed that providing time estimates of mortality risk is important, but most felt that estimates should be part of a broader discussion of the likely course of illness over the short and longer term. Patients described wanting information to understand the origin or cause of their liver disease, how to prevent complications, and tips for managing the condition to provide meaning and context for the prognosis discussions. For example, participants described wanting to know how the condition may affect their quality of life, including their mobility, change in diet and energy level, and other symptoms that accompany disease progression. Participants also described a psychosocial element involving coping with emotions from disbelief to acceptance, managing worry and anxiety, and guidance on how to manage the condition within one’s life.

Timing of prognosis discussions was another important theme raised by clinicians. Many clinicians endorsed having prognosis discussions early (before decompensation), especially those who view a more expansive role for such conversations. These participants supported revisiting prognosis discussions after decompensation or progression of the disease. Patients also endorsed earlier conversations. Clinicians who viewed prognosis conversations narrowly as discussions about mortality favored delaying discussions until after complications occur. Preserving hope and decreasing anxiety were reasons given for delaying prognosis discussions, but there is little evidence that prognostic disclosures diminish hope and may even provide hope when discussions are broader in scope [26].

Most clinicians assigned the primary responsibility for leading prognosis conversations to hepatology physicians. In addition to their authoritative role, participants deferred to these physicians due to their assumption of having rapport and relationships with patients. Some pointed to palliative care, but palliative care clinicians viewed their roles as supportive. In contrast, patients had the least preference for who should lead or participate in prognosis discussions. They are more concerned with the content of such discussions and the opportunity to ask questions.

Interviews suggest that clinicians prefer multi-professional teams for conducting prognosis discussions with different clinicians having specific roles and responsibilities. Prognosis discussions for AdvLD are conceptually similar to serious illness conversations [27]. Serious illness conversations express three key tasks: establishing connection, delivering information, and eliciting values, goals and preferences [27]. Triangulating our data from patients and their clinicians with this prior literature, we suggest the following approaches to prognosis discussions. We believe a variety of clinician-members from liver care teams (hepatology or gastroenterology physician, hepatology advanced practice provider, or hepatology social worker) with a personal relationship to the patient can initiate conversations. Clinicians with rapport can establish connection by looking for emotional cues, responding with empathic statements, and assessing whether the patient is ready to move forward [28]. Consistent with prior literature, we found that patients differed in what they wanted to know. Clinicians should ask what the patient understands about his/her condition, and how much he/she wants to know about the course of the illness [27]. Study participants describe effective discussions of prognosis as conversations that provide information as a range, acknowledge uncertainty about time estimates, include written materials with numbers and figures, and encompass estimates of disease course and potential complications [26, 27, 29], including mobility, cognition, and any bothersome symptoms. Patients also want information on specific self-care tasks they could do to improve their prognosis. The serious illness conversation literature also describes the importance of eliciting patient values, goals and care preferences within the context of limited prognosis [27]. As part of building connection during prognosis conversations, clinicians should ask about what matters most to the patient (values) and how values translate into specific, actionable goals for treatment [28]. In addition, clinicians should learn what patients are willing/not willing to do (preferences) to reach their outcome goals.

Prior studies describe the difficulties liver specialists have initiating prognosis discussions and making early referrals to palliative care and advanced care planning [5, 6, 8]. Low et al. proposed specialty liver clinics with palliative care involvement as a potential intervention for early prognosis discussions and supportive care. The findings of the current study build on this prior literature to provide further details of structure, contents and participants of a potential intervention. Table 2 summarizes these key components of AdvLD prognosis conversations integrated from our results and the prior literature: 1) building rapport; 2) discussing prognosis; 3) eliciting patient priorities, and 4) aligning care options to achieve priorities. The recommended activities and clinician responsibilities for each component are described. Table 2 also describes the timing and context of initial and subsequent prognosis discussions. Future research can determine how these additional features of serious illness conversations best integrate into AdvLD care. In the current study, clinicians expressed hesitation with prognosis discussion arising from unfamiliarity or a lack of training with prognosis discussions. Further development of the model proposed in Table 2 will require translation of the tips and scripts used for serious illness conversations developed for cancer care [30] and multimorbid older adults [30, 31] for the context of AdvLD. Future pilot studies of this model, including observational analysis of patient-clinician conversations, are necessary to test the effectiveness of some or all components proposed in Table 2.

**Table 2:** Prognosis discussions for an integrated approach to advanced liver disease care.

Initiate early prognosis discussions at the time of cirrhosis diagnosis (prior to onset of complications). Discussions include the following components and related activities. Roles and responsibilities shared among multiple clinicians.		
Components	Activities	Roles and Responsibilities
Building Rapport	Identify emotions, making a connection, ask about fears and worries, understanding desire for information	Member of the Hepatology team with personal connection to patient who can initiate prognosis discussions
Discussing Prognosis	Provide reliable time estimates for survival, communicate what to expect about course of illness, use numbers with ranges, include charts or figures	Member of the Hepatology team who understands prognosis models and can discuss illness trajectories
Identify patient priorities	Elicit what matters most (values), transform values into specific, measurable outcome goals (what do you want to achieve from your care), ask about care preferences (what is burdensome, what are you willing and not willing to do for self-care and treatment)	Member of the Hepatology team skilled at serious illness conversations and eliciting patient priorities (nursing, social work, advanced practitioners)
Align care to priorities	Identify the full, holistic range of care options for advanced liver disease (nutrition, physical therapy, palliative care, symptom management), develop and apply care pathways to aligning care options to achieve outcome goals	Hepatology clinicians, dieticians, physical therapists, social workers, palliative care, pharmacists, mental health, addiction specialists
**At each subsequent appointment**: Re-visit how well patients are achieving their identified priorities. Refine priorities if they are unrealistic or unclear. Adjust or change treatment plan to better align with the identified priorities.		
**After experiencing a cirrhosis complication:** Re-visit the full prognosis discussion (all four components). Clarify how the complication may have changed prognosis and how that might affect priorities.		

This study has limitations. We recruited clinicians and patients from three geographically dispersed VA health systems which limits the external validity of findings beyond similar VA sites. Moreover, we sampled a variety of clinicians who typically provide AdvLD care and may have skewed perspectives on prognostic conversations. As the majority of clinicians were female and all patients were male, participants’ gender may influence the prognosis discussions. It is possible that our sampling strategy did not render a sample of patients that represents the population of all people with advanced liver disease. The VA population is predominantly male, and all of our participants were males. We recruited patient participants seen in specialty liver clinics and their perceptions may not reflect liver disease care in other non-specialty settings or in non-veteran populations. The majority of the patients were Child-Pugh A and not experiencing liver-related symptoms; this could impact their opinions about prognosis discussions. We excluded individuals who were currently hospitalized, which may impact the interpretation of our results since prognosis discussions often occur in this setting. Although we measured patients’ education level, we did not assess their health literacy; health literacy may influence patients’ experiences in prognosis discussions. Further, the results of the study rely on participants’ recall of prior conversations rather than direct observation of patient-clinician conversations and immediate assessment. Our approach could introduce some retrospective recall bias. Finally, all participants spoke English; caring for a non-English-speaking patient population may require different educational materials as well as an in-person interpreter. While potential biases exist in all research, our approach to data collection, analysis, and reporting was rigorous. Non-clinician interviewers conducted interviews and analyzed data. Data were coded by three individuals who met frequently to discuss codes and enhance reliability. Finally, coding and emerging themes were discussed among members of a multidisciplinary research team.

## Conclusion

Discussing prognosis serves as a foundation for an integrated model of patient-centered AdvLD care [1]. Our data suggest that AdvLD prognosis discussions that begin early in the course of illness may be more beneficial. These early discussions could build rapport, establish clear expectations about the future, and prepare patients and caregivers to be participants in their care and treatment planning. Discussions about prognosis can be revisited given the uncertain course that most patients with AdvLD endure to prepare them for changes in mortality risks, symptom burden and self-care needs. Effective prognosis discussions include clear professional roles, lines of communication, mutual support, and situational monitoring to ensure a multi-professional approach to AdvLD conversations [32]. Coordinated prognosis conversations, along with registries that identify AdvLD status and trigger notifications to ensure timely scheduling of conversations with patients, may provide a framework for integrated, patient-centered AdvLD care. Our findings build on prior literature suggesting that this comprehensive approach to prognosis discussions for serious illnesses like AdvLD could be embedded within a broader framework for serious illness conversations and care planning. Future studies are needed to empirically evaluate this framework for AdvLD care.

## Supporting information

S1 Appendix Interview guides. (DOCX)

S2 Appendix Patient codebook. (DOCX)

S3 Appendix Provider codebook. (DOCX)
