# Supplementary material for: Prognosis conversations in advanced liver disease: A qualitative interview study with health professionals and patients
Source: PLoS One. 2022 Feb 18;17(2):e0263874. doi: 10.1371/journal.pone.0263874 (PMC8856527; doi:10.1371/journal.pone.0263874)
Supplement: S1 Appendix — (DOCX) [file pone.0263874.s001.docx]

# Healthcare Provider Interview Guide

“For this interview, I will be asking you some questions about your experiences caring for patients with advanced liver disease. You can skip any questions that you do not want to answer.”

“If you agree, your answers will be recorded using a digital voice recorder. Do you agree to be recorded?”

If yes: “Thank you. Once I start the recording, I will ask you to confirm that you have given your permission to be recorded so that I will have documentation of your agreement.”

If no: “Ok, that’s fine. I will take notes and will not record the interview.”

If the participant agrees to be recorded, start the recording and say:

“This audio recording will be used for this research study only, and will not be disclosed outside of the VA. The recording will be transcribed by a VA transcriptionist so that we can analyze what is said. Could you please confirm that you agreed to be recorded as part of a research study entitled *Patient centered care for individuals with advanced liver disease?* And that you agreed to answer questions about your usual practices managing patients with advanced liver disease?”

## Caring for AdvLD Patients

1. I’d like to know all about your experiences caring for patients with advanced liver disease.

- Tell me about your patients—who are they? What are their lives like?
- Do your patients have social support? How does this affect their experience with liver disease?
- How severe is their illness when they come to you?
- What effect does the stage of liver disease have on your experiences caring for these patients?
- What treatments do you offer your patients?
- Do you focus more on supportive care in some stages? Palliative care in some stages?

2. What do you think patients and caregivers need to know about advanced liver disease?

3. To what extent do you talk about preventing complications and managing complications?

- How is this information currently shared with patients?

(probe: videos, pamphlets, counseling, active teaching)

- In your setting, who has the responsibility of sharing that information with patients?

(probe: specific persons, staff, disciplines)

- How do you know whether patients understand the information?

4. Do you teach patient how to do self-care for advanced liver disease? What do you teach them?

- How is this information currently shared with patients?

(probe: videos, pamphlets, counseling, active teaching)

- In your setting, who has the responsibility of sharing that information with patients?

(probe: specific persons, staff, disciplines)

- How do you know whether patients understand and are able to do these self-care tasks?

5. To what extent do you talk to your patients about their health outcome goals?

- How do you discuss these health outcome goals with patients and their caregivers? Do you have explicit conversations about their health outcome goals (about how the disease affects the things they’d like to do in their life, things they want to be able to do)?
- Based on your experiences, what some common health outcome goals for patients with advanced liver disease? (probe: specific examples)
- Do patients share specific goals (be able to walk the dog twice a day), or do you usually talk about more global goals (like living longer)?
- In your experiences, how do patients’ health outcome goals change as their disease advances?

6. What strategies do you use to align a patient’s health outcome goals with their treatment plan? (probe: specific examples)

- For example, think about medication reconciliation. Do you talk about a specific medication and its purpose and whether the purpose relates to the patient’s health outcome goals?
- In what ways do you try to formulate treatment plans to achieve patients’ health outcome goals?
- What do you do in cases where the patient’s goals do not align with the treatment plan? For instance, what do you do if you realize that a patient is using a medication that the purpose does not align with their goals? (probe: specific examples)

## Discussing Prognosis

7. Describe how you share or discuss information with patients and their caregivers about the prognosis for their illness.

- How does the conversation go?
- Who usually brings up the topic?
- What resources and tools do you currently use to discuss prognosis?

8. What would you recommend for ways to improve how prognosis and risk information is shared with patients and caregivers?

- What resources could you use to improve the conversations you have about prognosis and risk information?
- Who needs to be part of the conversation? (spouse, caregiver)
- What staff should be involved in these conversations?

#### In later interviews:

9. Please look over the example health information we provided.

- How is this information valuable or helpful to you?
- In what ways is it confusing or unclear?
- What could make this information more useful to you?
- What other types of similar information would you want?
- Do you feel patients and caregivers could understand this information?

## Care across the Illness Trajectory

10. How do you distinguish between curative, supportive, and palliative care?

- In your opinion, is this a necessary/important distinction to make? Why?
- How could providers talk about this distinction to their patients in a way that is practical and meaningful to patients?

11. What therapeutic options are considered curative care for liver disease?

- What are the desired patient outcomes for curative care?
- When in the patient’s illness course do you discuss treatment options that are considered curative care?

12. What therapeutic options are considered supportive care? (symptom management—can go on a long time)

- What are the desired patient outcomes for supportive care?
- When in the patient’s illness course do you discuss treatment options that are considered supportive care?

13. What therapeutic options are considered palliative care? (end of life)

- What are the desired patient outcomes for palliative care?
- When in the patient’s illness course do you discuss treatment options that are considered palliative care?

14. When do you discuss end of life planning?

- What are the triggers for having this conversation?
- How do you bring up the conversation?
- If participant does not have these conversations: Who in the healthcare team typically has these conversations? How are they typically done?

## Integrated Care

15. How well integrated are curative, supportive, and palliative care in your current practice?

- In your view, what would a truly integrated approach to cirrhosis care look like?
- Is there a way to still distinguish between curative, supportive, and palliative care, but make a comprehensive and cohesive care program? How could this be achieved?

16. How would you be able to adapt your current workflow and routine to allow for a more integrated approach, like the one you described?

- What resources, staff, and leadership do you have in your setting that could facilitate a more integrated approach to cirrhosis care?
- What barriers do you face to adopting a more integrated approach?
- What additional resources would you need?

## Collaborative Treatment Planning

17. Describe your process for developing a patient’s treatment plan for advanced liver disease.

- What role do the patient and their caregiver have in this process? (probe for examples)
- What tools, resources, or staff are necessary to better engage patients and caregivers in this process?

18. How do you factor in patients’ goals in working with them to develop a treatment plan?

- How do you alter a patient’s treatment plan to facilitate their health outcome goals?
- What tools or materials do you use to do collaborative treatment planning?
- How do you introduce these materials to patients?

19. In your opinion, what does a truly collaborative approach to liver disease treatment planning look like?

20. Please take a look at the journey map.

- Where in the patient’s illness trajectory do you think a more collaborative approach can and should occur?
- Where in the patient’s illness trajectory would you discuss different types of prognosis data? What are the different types of prognosis information that is best suited for various times along the illness trajectory?

21. What would facilitate a more collaborative approach to treatment planning? (context, procedures)

- What barriers do you encounter in using a collaborative approach to treatment planning?
- What skills and resources do you think you would need?
- What additional staff or health care professional are needed?
- How would their roles and responsibilities differ from yours?

22. How useful do you think it would be to have decision support tools to assist in conversations with liver disease patients?

- What kinds of tools would be most useful?
- What is the best way to implement decision support tools into the routine process of liver disease care?

# Patient Interview Guide

“For this interview, I will be asking you some questions about your firsthand experiences of living with liver disease. I will also ask you some questions about your experiences receiving care and planning treatment at the VA for your liver condition. You can skip any questions that you do not want to answer.”

“If you agree, your answers will be recorded using a digital voice recorder. Do you agree to be recorded?”

- If yes: “Thank you. Once I start the recording, I will ask you to confirm that you have given your permission to be recorded so that I will have documentation of your agreement.”
- If no: “Ok, that’s fine. I will take notes and will not record the interview.”

If the participant agrees to be recorded, start the recording and say:

“This audio recording will be used for this research study only. The recording will not be disclosed outside of the VA. The recording will be transcribed (typed word-for-word) by a VA transcriptionist so that we can analyze what is said. Could you please confirm that you agreed to be recorded as part of a research study entitled *Patient centered care for individuals with advanced liver disease?* And that you agreed to answer questions about care you have received for your liver condition?”

## Living with Liver Disease

1. I’d like to hear all about your experience with having liver disease.

- Can you take me back to the beginning?
- When did you first learn that you have liver disease?
- What was going on in your life at the time?
- How did you come to have liver disease?
- How did you feel when you learned you had liver disease?
- Can you tell me what it has been like living with liver disease?
- What impact has it had on your life?

(probe: fatigue, pain, mood, physical limitations, other symptoms; feeling about yourself)

- How do you manage this illness? What do you do to take care of it, and yourself?
- Do you have people in your life to help you manage your illness?
- How does this illness affect others in your life, or how you relate to them?
- What other significant changes have there been in your life in the past few years?

2. How serious do you think your illness is? How is your illness affecting your quality of life right now?

- What do you think might happen over the next few months or years because of your illness?
- Do you have concerns about your quality of life in the future?

#### Values

3. I want to know more about you and what is important to you. What makes you happy? I know you have liver disease and it probably keeps you from doing some things you enjoy or spending time with people who are important to you. Can you tell me about some of those relationships and activities that matter most to you? What is most important to you? If your health didn’t get in the way, what would you be doing more of?

(probe value domains: CONNECTION: personal relationships, social connections, spiritual needs; LIFE ENJOYMENT: hobbies or other enjoyable activities, work or other meaningful vocations; FUNCTIONING: independence, daily functioning, needing help from others; MANAGING HEALTH: physical or emotional symptoms, concern about your health or life)

#### Common Symptoms & Barriers

4. How has your illness gotten in the way of the things that matter most to you? What are the barriers that keep you from [restate their values]?

(probe: fatigue, pain, physical limitations, etc.)

## Information Sharing

#### Prognosis

5. Tell me what you know about your illness. What is your understanding of liver disease and what is happening to your body?

6. What has your health care provider told you about your liver disease?

(probe: cause, severity, prognosis)

7. How did they share this information with you?

- How did you feel when they shared this information with you?

(probe: anger, sadness, concern, fears, any sense of relief, clarity, sense of understanding)

- Was this information easy or hard for you to understand?
- Is there anything you would change about the type of information your health care provider shared?
- Is there anything you would change about how they shared this information?
- Would you change anything about when (timing) they shared this information?

8. Thinking about what the doctor told you, how did this change your perspective about your illness? Did it change your day to day life? How you might want to live your life?

#### Treatment Options

9. What has your provider told you about available treatment options?

- Have you talked about treatments to reverse the disease?
- Have you talked about managing symptoms and preventing complications?

10. How did your provider share this information?

- How did these conversations with your doctor make you feel?
- Was this information easy or hard for you to understand?
- Is there anything you would change about how this information was shared?
- Would you change anything about when (timing) they shared this information?
- Would it be helpful to talk about treatment options available at different stages of the disease? (ex. What treatments would you do to cure; what treatments would you do to prevent complications, what treatments would you do to manage symptoms?)

#### Learning Goals

11. What don’t you understand about liver disease that you want to know more about? What are you uncertain about? Is there anything you are worried about that you might want to talk to your doctor about?

(probe: what to expect, how to prepare, how to prevent complications, how to handle complications)

- How would you like to receive this information?

(probe: different methods, timing, involvement of loved ones, involvement of different healthcare providers)

***In later interviews:***

The Qualitative Interviewer will skip Question 12 for the 1^st^ five Houston interviews, as they are planned to occur prior to completion of Aim 1 risk prediction model development.

12. Please look over the health information we provided. This is a summary of your health information.

- What does this information mean to you?
- How is it helpful?
- How is it unhelpful?
- How does this information make you feel?

## Experiences of Care

#### Care Preferences

13. Tell me about your current treatment plan.

14. What do you think of your current treatment plan? How is treatment going for you?

(probe: symptoms, psychosocial needs, functional limitations, appearance, pain)

15. What parts of your treatment are a burden? Are there parts of your treatment that you don’t always do because they are burdensome, difficult, or uncomfortable?

(probe: medications, tests, visits, procedures)

16. Has your provider recommended treatments/care that you are not willing to try?

- Why did you decide to not try that?

17. What parts of your treatment plan are working well? (probe: medications, lifestyle modifications)

- What parts of your treatment do you feel confident about? How did you become confident?

(probe: instruction/demonstration from a provider/nurse?)

#### Treatment Planning

18. How involved were you in making decisions about treatment for your liver disease?

19. How involved was your caregiver?

20. In what ways would you and your caregiver like to be involved in making treatment decisions?

## Health Outcome Goals

21. Before, we talked about the things in life that are most important to you. You mentioned [restate their values] and you also said that [restate barriers] get in the way of doing these things. Have you thought about or talked with your provider about ways to address or treat those parts of your illness?

22. Sometimes patients have certain goals for their treatment, so I’d like to talk to you about your treatment goals. These are the things that you will do when you feel well. Examples: working in the garden twice a week; going out to lunch with friends every weekend; taking the dog for a walk around the block twice a day.

- What are some of your treatment goals—those things that you want for your treatment to allow you to be able to do?

23. Which of these goals that you just mentioned are most important to you? Tell me about why these goals are important to you.

24. Is your treatment plan helping you to be able to do these things?

- If no: What are the symptoms/barriers that your treatment plan isn’t addressing well enough? (probe: fatigue, pain, physical limitations, etc.)

25. Have you talked with your health care provider about these things that you want to do? Have you discussed that it is important to you to be able to [restate their goals]?

- Can you tell me about some of those conversations? How they went? How you felt?

26. Is your caregiver/significant other aware of your goals?

- Can you tell me about the conversations you have had with them about your goals?

27. How might your goals change as your illness progresses?

(probe: quality of life vs. quantity: will they start to focus more on immediate comfort rather than possibly undergoing discomfort to maximize longevity)

#### Wrap Up

28. Is there anything else you’d like to share about your experiences with liver disease or liver disease treatment or the care you’ve received at the VA?
