# Supplementary material for: Prognosis conversations in advanced liver disease: A qualitative interview study with health professionals and patients
Source: PLoS One. 2022 Feb 18;17(2):e0263874. doi: 10.1371/journal.pone.0263874 (PMC8856527; doi:10.1371/journal.pone.0263874)
Supplement: S2 Appendix — (DOCX) [file pone.0263874.s002.docx]

**Advanced Liver Disease PATIENT interviews: Coding Scheme**

**DIAGNOSIS**

**Disease identification:** Descriptions of how/when patient first learned of liver disease diagnosis

**Reaction to diagnosis:** Comments about the patient’s reaction to diagnosis

Reaction to diagnosis caregiver: comments about how family and friends reacted to diagnosis

_____________________________________________________________________________________

**PERCEPTIONS OF HEALTH AND LIVER DISEASE**

**Cause of liver disease patient**: Comments about patient’s understanding of how they came to have liver disease

**Cause Hep C:** comments about Hep C infection resulting in liver disease

**Cause unknown:** comments about unknown cause of liver disease

**Cause alcohol abuse:** comments about attributing alcohol abuse to liver disease

**Cause of liver disease provider:** comments about how providers explained to patients how they came to have liver disease

**Perceived severity:** comments about their perception about severity of liver disease and why they think this

**Beliefs/understanding:** comments about their beliefs and understanding of what liver disease is, what it is doing to their bodies, and how the liver functions

**Missing knowledge:** information that patients wish they knew about living disease or aspects of liver disease that are unclear to them

**Expected disease trajectory:** comments about what patient expects to happen in the future due to liver disease

**End of life**: concerns patient has about end of life

**End of life communication:** communication with providers about end of life that they may have had or would like to have

**Health philosophy:** comments about their general approach to their health and any illness/disease they experience

**Trust in VA:** comments about how the Veteran trusts the VA to take care of them (or in some instances, has doubts about this); may also include general skepticism or confidence in their providers at the VA

_____________________________________________________________________________________

**ILLNESS EXPERIENCE**

**Treatment plan:** what they’re doing to take care of their health; also comments about whether or not treatment plan is meeting their needs and goals

**Treatment plan meaning**: how patients understand the meaning of treatment plan

**Treatment plan willingness:** comments about the extent to which the patient is willing to participate in or certain treatments or treatment plans

**Quality of life and limitations:** how is their health affecting their life

**Complications:** current or previous complications that the patient may have experienced

**Comorbidities**: existing physical and chronic diseases patient is also living with

**Hospitalization:** comments about hospitalization due to liver disease or other factors

_____________________________________________________________________________________

**LIVER TRANSPLANT**

**Liver transplant:** comments about liver transplant possibility and desirability; concerns they may have about liver transplantation

_____________________________________________________________________________________

**COMMUNICATION AND INFORMATION**

**Communication prognosis:** comments about what provider has said to them about prognosis

**Prognosis tools:** tools providers may have used to communicate information about prognosis

**Prognosis communication needs:** information patients would like to have about prognosis

_____________________________________________________________________________________

**HEALTH VALUES AND GOALS**

**Values and Goals:** what patients identify as being important to them and what they strive to do

**Communication values goals:** communication with providers about health outcome goals

**Goal change:** comments about whether or not their goals will change if their condition worsens

**Prioritization:** comments about whether patients prioritize quality over quantity and why

**Health Philosophy:** comments that indicate/describe a general approach of the patient to his health and healthcare treatment. Quotations assigned this code typically involve the patient describing a matter-of-fact approach to their healthcare and well-being and a sense that “what will be will be.”

_____________________________________________________________________________________

**CAREGIVERS**

**Caregivers/social support:** comments about social and caregiver support or lack of and need for/not
