# Supplementary material for: Prognosis conversations in advanced liver disease: A qualitative interview study with health professionals and patients
Source: PLoS One. 2022 Feb 18;17(2):e0263874. doi: 10.1371/journal.pone.0263874 (PMC8856527; doi:10.1371/journal.pone.0263874)
Supplement: S3 Appendix — (DOCX) [file pone.0263874.s003.docx]

**Advanced Liver Disease PROVIDER interviews: Coding Scheme**

**PROVIDER CHARACTERISTICS**

**Role Description**: Description of job role, e.g. Social worker, physician, nurse

**PATIENTS**

**Patient Panel—Health status:** Comments about the condition of patients when the interviewee/provider begins treating them

**Patient Needs—Financial:** Comments on specific needs to patients related to finances

**Patient Need—Caregiver Support:** Comments on specific needs of patients related to caregiver support

**Patient Needs—Further Education:** Comments on need for additional patient education around advanced liver disease.

**Patient Needs—Social and Mental Health Support**: Support for substance abuse, other social problems. This includes references to extent to which patients have social support and the importance of social support.

**Patient Knowledge Gaps:** Comments about areas where patients lack knowledge about their condition and treatment for the condition

**Patient Needs—Other**: Any additional needs not covered by other codes.

**Liver Transplant Patients:** Comments referring to unique needs/circumstances of liver transplant patients; how they differ from other advanced liver patients

**PROVIDER BELIEFS and EXPERIENCES**

**Definition Supportive Care:** How interviewee defines and describes supportive care

**Definition Curative Care:** How interviewee defines and describes curative care

**Definition Palliative Care:** How interviewee defines and describes palliative care

**PROVIDER COMMUNICATION**

**Provider Communication—Managing Disease:** Comments about how provider communicates with patients about managing symptoms and any complications that may arise

**Provider Communication—End of life**: Comments about communicating with patients around end of life planning and care, including the nature of these conversations, how they are broached, and challenges that might come up.

**Provider Communication—Caregiver**: Comments about how the provider communicates with the patient’s caregiver

**Provider Communication—Patient Education Tools:** Discussion of patient education tools the provider might use; recommendations for tools that might be effective

**Provider Communication—Health Outcome Goals:** Comments about provider communication with patient about outcome goals. Also includes mention of specific goals that patients might have.

**Provider Communication—Prognosis:** Comments about the extent to which provider communicates with patient and caregivers about prognosis; also includes timing of these conversations, challenges to having these conversations, and how prognosis conversations could be improved.

**Provider Communication—Curative Care:** Comments about provider communication with patients about curative care

**Provider Communication—Palliative Care:** Comments about provider communication with patients about palliative care. Includes barriers to discussion about palliative care

**Provider Communication—Supportive Care**: Comments about the characteristics of communication with patients around supportive care, including timing of, and content of conversations.

**Provider Communication—Self Care:** Description of how/when interviewee talks about self care

**Provider Communication—General Barriers:** Comments about barriers to communication more generally

**Provider Communication—Improvements:** Comments about ways that can improve patient communication

**INTEGRATED CARE**

**Integrated Care:** Comments about integrated care at the facility, including the current state of integrated care, ideal integrated care, challenges to integrated care, and resources needed to achieve ideal care.

**COLLABORATIVE TREATMENT PLANNING (CTP)**

**CTP Planning Process:** Discussion around provider’s use of CTP and what the process looks like

**CTP—Definition:** how the interviewee defines CTP

**CTP—Tools/resources needed:** tools the interviewee has identified as needed for CTP

**CTP—Barriers:** Barriers to engaging in CTP
